# The Good, the Bad, and the Ugly of ROS: New Insights on Aging and Aging-Related Diseases from Eukaryotic and Prokaryotic Model Organisms

**DOI:** 10.1155/2018/1941285

**Published:** 2018-03-18

**Authors:** Ana L. Santos, Sanchari Sinha, Ariel B. Lindner

**Affiliations:** ^1^Institut National de la Santé et de la Recherche Médicale, U1001 & Université Paris Descartes, Sorbonne Paris Cité, Paris, France; ^2^Defence Institute of Physiology and Allied Sciences, DRDO, New Delhi, India

## Abstract

Aging is associated with the accumulation of cellular damage over the course of a lifetime. This process is promoted in large part by reactive oxygen species (ROS) generated via cellular metabolic and respiratory pathways. Pharmacological, nonpharmacological, and genetic interventions have been used to target cellular and mitochondrial networks in an effort to decipher aging and age-related disorders. While ROS historically have been viewed as a detrimental byproduct of normal metabolism and associated with several pathologies, recent research has revealed a more complex and beneficial role of ROS in regulating metabolism, development, and lifespan. In this review, we summarize the recent advances in ROS research, focusing on both the beneficial and harmful roles of ROS, many of which are conserved across species from bacteria to humans, in various aspects of cellular physiology. These studies provide a new context for our understanding of the parts ROS play in health and disease. Moreover, we highlight the utility of bacterial models to elucidate the molecular pathways by which ROS mediate aging and aging-related diseases.

## 1. Introduction

Aging is characterized by a gradual loss of fitness over time. Aging is manifested as a series of dynamic changes at the molecular and macromolecular level over the course of a lifetime [1]. Faulty regulation of cellular processes can damage the cell's physiological integrity and subsequently lead to accumulation of damaged byproducts. Mankind has been fascinated with obtaining a better understanding of aging for many centuries, yet the exact mechanisms underlying the human aging process remain largely unclear. The aging process itself is complex due to several confounders, such as environmental factors, socioeconomic status, physical characteristics, and lifestyle [2].

Over the past few decades, life expectancy has increased linearly worldwide to an average of 60 years. The world's population over 60 is expected to increase from approximately 900 million people (12%) in 2015 to approximately 2 billion people (22%) in 2050 [3]. This increased life expectancy is associated with a reduced rate of child mortality, improved standards of living, and medical advancements, among others. Despite an increase in overall lifespan, aging and age-related diseases are major causes of mortality and morbidity worldwide [4]. Moreover, age-related disorders, such as Alzheimer's disease, dementia, cardiopulmonary disorders, diabetes, neurodegenerative and cognitive impairments, fragile physical condition, and psychosomatic disorders, are major causes of disability worldwide. These disorders account for over 20% of years lived with a disability [5]. Understanding the molecular mechanisms of aging is critical for developing therapeutic interventions that promote healthy aging.

**Figure 1:**
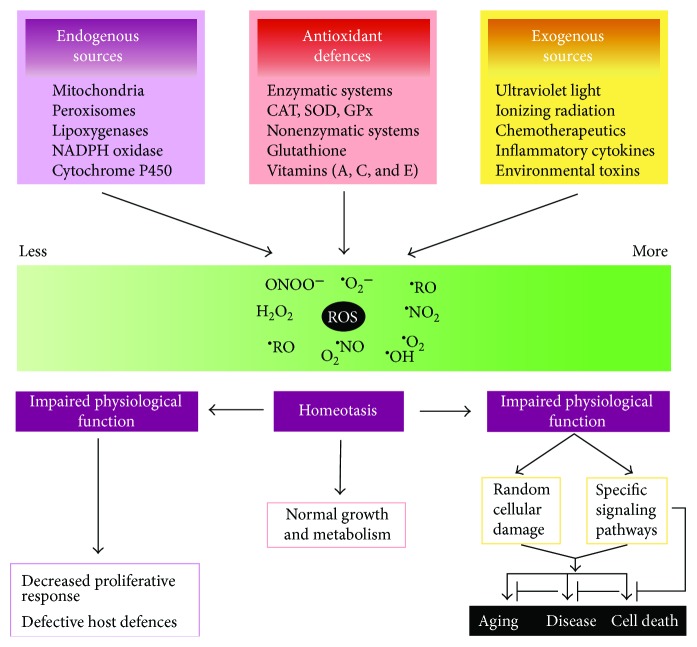
The sources and cellular responses to reactive oxygen species (ROS). Oxidants are generated as a result of normal intracellular metabolism in mitochondria and peroxisomes, as well as from a variety of cytosolic enzyme systems. In addition, a number of external agents can trigger ROS production. A sophisticated enzymatic and nonenzymatic antioxidant defense system including catalase (CAT), superoxide dismutase (SOD), and glutathione peroxidase (GPx) counteracts and regulates overall ROS levels to maintain physiological homeostasis. Lowering ROS levels below the homeostatic set point may interrupt the physiological role of oxidants in cellular proliferation and host defense. Similarly, increased ROS may also be detrimental and lead to cell death or to an acceleration in aging and age-related diseases. Traditionally, the impairment caused by increased ROS is thought to result from random damage to proteins, lipids, and DNA. In addition to these effects, a rise in ROS levels may also constitute a stress signal that activates specific redox-sensitive signaling pathways. Once activated, these diverse signaling pathways may have either damaging or potentially protective functions. Reproduced with permission from T. Finkel and N.J. Holbrook: Oxidants, oxidative stress and the biology of aging. *Nature*, vol. 408, no. 6809, pp.239-247, 2000.

Mitochondria often termed “the powerhouse of the cell,” metabolize carbohydrates and fatty acids via oxidative phosphorylation. Through this process, the mitochondria can generate 32 to 34 adenosine triphosphate (ATP) molecules per molecule of glucose. The protein complexes in the inner mitochondrial membrane collectively form the mitochondrial electron transport chain (ETC), which releases free radicals as byproducts of energy metabolism [6]. Harman originally proposed the free radical theory of aging in 1956 [7], according to which reactive oxygen species (ROS) are the primary mediators of the aging process. A brief overview of the sources of ROS and subsequent cellular responses is provided in Figure 1. The sources of ROS, antioxidant defenses, and subsequent biological effects have been reviewed elsewhere (e.g., [8]) and will not be covered in depth in this review. While extensive evidence indicates that enhanced ROS production and decreased ROS-scavenging ability shortens lifespan [9, 10], the free radical theory of aging has faced opposition, undermining the idea that ROS alone are responsible for the aging process. For instance, organisms can live a healthy lifespan in the absence of ROS scavengers [11–14]. Further, nutritional, pharmacological, and genetic interventions that increase production of ROS can promote longevity by activating mitochondrial oxidative phosphorylation and triggering downstream signaling pathways that promote an adaptive response [11, 15, 16], while pharmacological interventions that limit ROS production have been shown to shorten lifespan [11, 15].

While ROS and ROS-induced oxidative damage may not be the sole cause of the aging process, it is fairly consensual that ROS do play an important role in the molecular mechanisms that influence longevity. Thus, bridging the gap between the free radical theory and the current aging knowledge can help us to better understand how the interaction between ROS-induced oxidative damage and cellular metabolism affects aging and uncover genetic and pharmaceutical interventions that could modulate this interaction.

## 2. The Free Radical Theory of Aging and Beyond

Over the last few decades, the dominant aging model has been the free radical theory of aging. This theory states that organisms age because they accumulate oxidative damage produced by ROS. ROS are partially reduced metabolites of molecular oxygen generated by various metabolic reactions and cellular processes, such as respiration [11, 15]. Several studies support the free radical theory of aging. For instance, the garlic-derived thioallyl compounds S-allyl cysteine and S-allylmercaptocysteine have been shown to reduce ROS accumulation and increase *C. elegans* lifespan [17]. Similarly, treatment of *C. elegans* with four synthetic stilbene derivatives extended longevity by reducing ROS accumulation and oxidative stress [18].

However, recent research indicates that ROS play a more complex role in determining longevity than previously thought. For instance, *C. elegans* mutants lacking superoxide dismutase (SOD)—an enzyme that neutralizes the superoxide radical—while being more susceptible to multiple stressors, retain a normal lifespan [12]. In another study, deletion of the mitochondrial superoxide dismutase *sod-2* was actually found to extend the lifespan of *C. elegans* [19]. Furthermore, *C. elegans* lacking functional genes for subunits of the mitochondrial respiratory chain complexes I and III produce higher levels of superoxide, but they also have an extended lifespan. The extended lifespan of these knockouts can be completely abolished by treating them with the superoxide scavenger *N*-acetylcysteine [20]. Additionally, when wild-type *C. elegans* and the long-lived *clk-1* mitochondrial mutant were treated with paraquat, a superoxide generator, both the mean and maximum lifespan increased significantly [20].

### 2.1. Antioxidant Enzymes: Good or Bad?

Antioxidant enzymes play a key role in the neutralization of various ROS. However, the relationship between antioxidant enzyme levels and lifespan is not straightforward.

Several studies investigating the role of the antioxidant defense system in regulating longevity have shown that increased resistance to oxidative stress can improve longevity in mice [21, 22]. For instance, Cu/Zn superoxide dismutase 1 knockout (*Sod1*^−/−^) mice have significantly decreased lifespans. This reduced lifespan was associated with increased cellular senescence based on the increased expression of the senescence markers p16 and p21 [23]. Further, mitochondrial catalase overexpression has been connected to the increased median and maximum lifespan in transgenic mice overexpressing peroxisomal, nuclear, and mitochondrial catalases [24]. Mitochondrial catalase overexpression has also been shown to reduce various age-related pathological conditions, such as cardiac problems, inflammation-related disorders, and cancer [25].

However, other studies have found that increased antioxidant enzyme activity does not contribute to extended lifespan in rodents [26]. For instance, a study of transgenic mice overexpressing Cu/Zn SOD, Mn-SOD, and catalase, either alone or in combination, showed that overexpression of these enzymes did not significantly improve longevity compared with wild-type (WT) mice [27].

Glutathione peroxidase 1 (GPX1), the main isoform of the GPX protein family, is an important antioxidant enzyme that is ubiquitous in cells and plays an important role in the neutralization of hydrogen peroxides. While GPX1 expression has a protective effect against ROS-mediated cellular damage, *Gpx1*-knockout mice showed no evidence of increased oxidative damage to proteins and lipids, compared with their WT littermates [28]. By contrast, mice lacking both Mn-SOD and *Gpx1* had a higher level of oxidative DNA and protein damage, but their lifespan was not reduced compared with WT littermates [29]. Moreover, single-nucleotide polymorphisms of Mn-SOD and *Gpx1*genes have been shown to impact aging and longevity [30].

Another GPX family protein, GPX4, plays a major role in protecting the plasma membrane from peroxide-induced lipid damage. Null mutations of the *Gpx4* gene are lethal in mice. Ablation of GPX4 in a transgenic mice line (C57BL/6 background) resulted in increased oxidative damage in the brain as well as neuronal loss compared with WT mice [31]. Transgenic overexpression of GPX4 was shown to protect mice from the lethal null-mutation phenotype and prevented oxidative-stress-induced liver damage and cell death [32]. However, mice with reduced GPX4 expression and activity showed no significant differences in mean, median, and maximal lifespan compared with WT mice [33].

Thioredoxin (Trx) is a redox protein that acts as a hydrogen donor in many reductive reactions in cells. It has two forms: cytoplasmic (Trx1) and mitochondrial (Trx2). Similar to *Gpx4*, *Trx2* null mutations are lethal in mice [34], and *Trx2* knockout impairs mitochondrial function by decreasing ATP production; increasing ROS production; inducing oxidative DNA, protein, and lipid damage in the liver; and increasing oxidative-stress-induced apoptosis of liver cells [35]. Trx1 overexpression (Tg(*TRX1*)^+/0^) has been shown to protect against oxidative damage of cellular macromolecules and extend the earlier part of the lifespan in male mice; however, neither male nor female Tg(*TRX1*)^+/0^ mice showed changes in maximum lifespan [36].

The cellular location of ROS production may determine whether ROS play a beneficial or detrimental role. For instance, deletion of mitochondrial *sod-2* in *C. elegans* has been shown to promote longevity, whereas deletion of cytoplasmic *sod-1* and *sod-5* limits lifespan [37]. ROS produced by mitochondrial respiratory complex I reverse electron transport have been shown to improve lifespan in *Drosophila* [38]. Moreover, respiration inhibition appears to activate the hypoxia-inducible factor-1 (HIF-1) by elevating ROS levels. This activation has been shown to increase longevity [17, 18]. Studies in genetically modified mice have shown that a moderately impaired mitochondrial function can result in healthier aging, whereas severely altered mitochondrial homeostasis can be detrimental [39, 40]. Based on these observations, it is clear that both the level and location of ROS production contribute to determining the role of ROS in regulating longevity [41].

## 3. Role of ROS in Nuclear and Mitochondrial DNA Damage

Nuclear and mitochondrial DNA damage caused by ROS contributes significantly to the aging process. Under normal physiological conditions, a myriad of DNA repair mechanisms work in harmony to keep damage contained. Base excision repair, mismatch repair, nucleotide excision repair, and double-strand-break repair all work rigorously to mend DNA damage induced by ROS, X-rays, UV and ionizing radiation, alkaline agents, replication errors, antitumor agents, and various chemical agents [42]. Deficiencies in any of these repair mechanisms can accelerate the onset of aging [43].

The DNA theory of aging, first postulated by Szilard in 1959 [44], correlates the steady accumulation of DNA damage with imbalances in cellular function, ultimately leading to cell and organismal aging. Vilenchik and Knudson [45] calculated that the mammalian genome can sustain as many as 1000 lesions per hour per cell. These lesions include oxidative damage to bases, cross-linkages, and single-/double-strand breaks. Endogenous ROS usually cause the formation of abasic sites by breaking the glycosidic bonds between nucleotide bases and deoxyribose residues [46, 47]. Environmental agents like UV rays and chemical mutagens cause strand breaks through base modifications and intercalations [48, 49]. When unrepaired damage accumulates, it triggers the DNA damage response (DDR) [50, 51], which activates DNA repair systems [43]. Despite the number of lesions from which the genome suffers, the frequency of actual mutations is much lower, precisely because of these well-coordinated sensing and repair systems. However, when DNA repair mechanisms are overwhelmed or become dysfunctional, the DDR triggers senescence or apoptosis to suspend or eliminate the damaged cells, respectively. The accumulation of senescent cells in aging tissues [32] has been implicated as the driving force in the aging process, primarily through inflammatory pathways [33].

DNA repair can be divided into three types: base excision repair (BER), nucleotide excision repair (NER), and nonhomologous end joining (NHEJ). These processes have been reviewed exhaustively in the literature [52–54]. BER typically repairs oxidative DNA damage, most commonly the 8-oxoguanine lesion [55]. Briefly, DNA glycosylases excise the damaged base and a polymerase inserts the correct nucleotide in its place [56]. NER corrects more complex lesions not associated with oxidative damage, such as adduct formation between bases and UV-ray-induced cross-linkages [57]. While excision repairs primarily occur during replication, NHEJ can repair DNA double-strand breaks during the resting state as well [58]. NHEJ is a 3-step process that starts with the binding of the broken strand end to the Ku protein. The damaged and/or mismatched nucleotides are then removed, and the correct sequence is synthesized by DNA polymerase [59].

Unsurprisingly, studies have observed an age-related decline in DNA repair protein levels and activities [55]. Reduced BER activity has been reported in different tissues in older humans [60] and in mice lacking sirtuin 6, a histone deacetylase that is active during DNA repair [61]. Decreased levels of Ku protein and other NHEJ mediators are seen during normal human aging and in cases of Alzheimer's disease [62]. Similarly, NHEJ activity also decreases in aged rats that have accumulated DNA strand breaks in their neurons [63].

**Figure 2:**
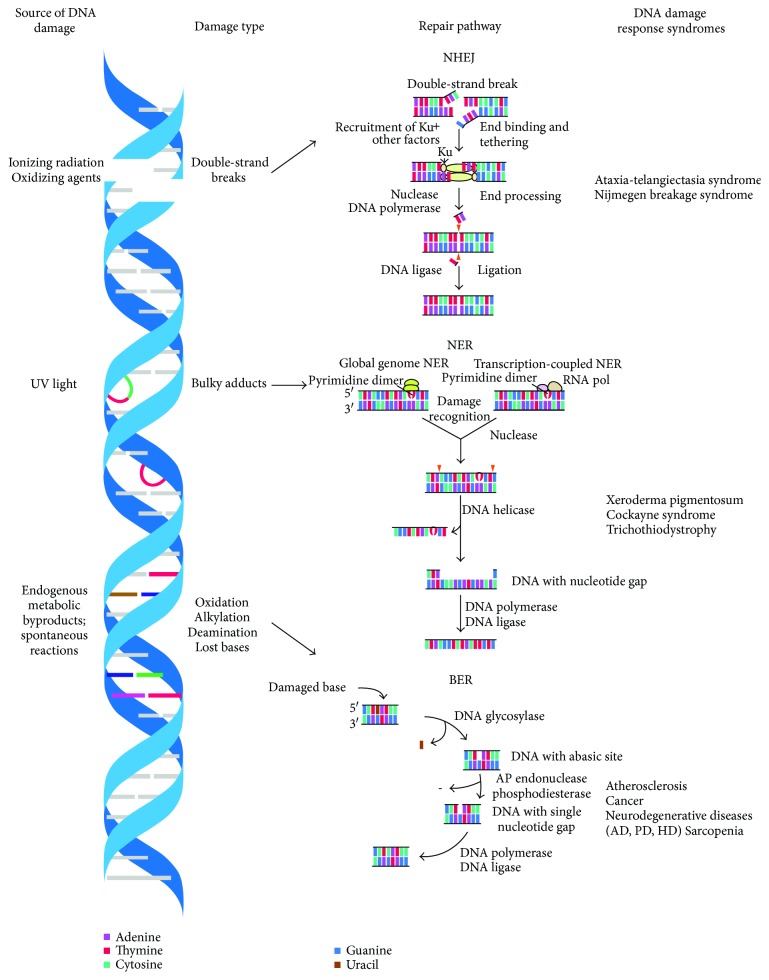
Examples of distinct DNA damage repair and response defects leading to genetic disorders in humans. Various damage types, including DNA double-strand breaks, bulky lesions, and base lesions, require nonhomologous end joining (NHEJ), nucleotide excision repair (NER), and base excision repair (BER), respectively. Defects in DNA-damage-response pathways lead to genome instability and, consequently, to complex syndromes characterized by tissue degeneration, cancer susceptibility, developmental defects, and premature aging. AD: Alzheimer's disease; PD: Parkinson's disease; HD: Huntington's disease.

The strongest evidence for the DNA theory of aging comes from human progeroid (i.e., premature aging) syndromes, such as Werner syndrome (WS), Bloom's syndrome (BS), and xeroderma pigmentosum (XP). These syndromes are caused by genomic instability and an underlying defect in DNA repair. WS and BS are caused by loss-of-function mutations in the *WRN* and *BLM* genes, respectively [64, 65]. These genes encode RecQ helicases, which are involved in both DNA replication and repair and are known to interact with the Ku protein [66, 67]. Murine knockouts of WRN and BLM have significant genomic instability and impaired DNA repair mechanisms compared with WT mice [68, 69]. XP is characterized by a mutation in the excision repair cross-complementation group 1 xeroderma pigmentosum group F (ERCC1-XPF) nuclease, which plays an important role in both NER and NHEJ repairs [70]. Mice lacking *ERCC1* show accelerated skin aging and increased DNA damage and cellular senescence compared with WT mice [71]. Replicative telomere shortening has been implicated in aging based on studies in the telomerase-knockout mouse model. This mouse model exhibits progeria and accumulates extensive DNA damage (reviewed by [72]). Telomere shortening also accompanies human progeria syndromes, such as WS and BS. More recent studies have directly linked defective DNA repair—specifically BER and NER—to the sites of telomere-uncapping-induced DDR [73, 74]. Examples of specific DNA damage repair and response defects that lead to genetic disorders in humans are shown in Figure 2. Thus, there is substantial evidence linking impaired DNA repair with aging syndromes; however, further studies are needed to provide a direct mechanistic link.

Since mitochondria are the main sites of ROS production, mitochondrial DNA (mtDNA) contains higher levels of oxidative damage and its mutation rate is significantly greater than that of the nuclear DNA [75]. In addition to their proximity to the sites of ROS generation, it is likely that the mitochondrial genomes are more prone to oxidative damage because histones and other chromatin-associated proteins, present in nuclear genomes where they act as scavengers of oxygen radicals, are absent in the mitochondria. The existence of repair of oxidative damage to mtDNA, originally reported in the early '90s, is well established [76–78]. BER appears to be the only excision repair process active in the mitochondrial genomes. All mtDNA repair proteins are encoded by the nuclear genome and imported into the mitochondrial matrix. Most mtDNA repair proteins discovered so far are isoforms of the nuclear BER proteins arising from differential splicing or truncation of the terminal sequences [79, 80]. The mitochondrial DNA polymerase *γ* (Pol*γ*) and mtDNA ligase (Lig III*α*), involved in mtDNA replication, appear to also be functional in mitochondrial BER [79, 80].

Accumulation of somatic mtDNA mutations has been found to accelerate normal aging [81–84], leading to oxidative damage, energy failure, increased production of ROS, and accumulation of amyloid-beta peptide (Abeta) [85, 86], a key molecule in Alzheimer disease (AD) [83]. A vicious cycle ensues which reinforces mtDNA damage, the impairment of the mitochondrial respiration, and oxidative stress.

## 4. Role of ROS in Protein Homeostasis

Similar to DNA damage, age-related protein damage and the accumulation of damaged protein products contribute to aging. Therefore, it is critical to understand how ROS contribute to an imbalance in cellular protein homeostasis and alter the aging process.

Free radicals can “attack” proteins, causing oxidative damage. Oxidative damage can alter protein function. Further, it can produce carbon-oxygen double bonds at arginine, lysine, proline, and threonine side chains, forming reactive ketones or aldehydes, known as protein carbonyls [87], normally considered to reflect the overall levels of cellular oxidative stress [88]. Protein carbonyls are associated with the production of aberrant protein isoforms [89, 90]. Unlike other oxidative modifications, such as disulfide bond formation, protein carbonylation is irreversible. Thus, the only means of limiting the damage caused by the affected proteins is their degradation. As more oxidative damage accumulates, proteins are more likely to misfold. Moderately oxidized proteins undergo degradation by the proteasome, the highly sophisticated protease complex designed to carry out selective, efficient, and processive degradation of short-lived, damaged, misfolded, or otherwise obsolete proteins [53]. However, heavily oxidized proteins can crosslink with other proteins, which prevents their degradation [54]. As a consequence, heavily damaged proteins accumulate within the cell, affecting its proper functioning. Accordingly, impaired proteostasis is a hallmark of many age-related diseases, including Alzheimer's and Parkinson's disease [91, 92].

Many studies have shown links between protein homeostasis, ROS, and oxidative stress. For instance, reducing insulin/IGF-1 signaling or inhibiting downstream mTOR signaling has been shown to improve the homeostasis of Alzheimer's disease-associated proteins, promoting longevity and protecting cognitive function in animal models [93]. Several studies in *C. elegans* have also shown that the heat shock factor 1 (HSF-1) works with the FOXO-like transcription factor, *daf-16*, to improve protein homeostasis and increase lifespan [94, 95]. Treating *C. elegans* with the amyloid-binding dye thioflavin T has been shown to reduce protein aggregation and extend lifespan via HSF-1- and SKN-1-/Nrf-mediated signaling [96]. Another study comparing the role of small heat shock proteins in *Drosophila* identified two proteins—CG14207 and HSP67BC—involved in proteostasis which mildly improved longevity when overexpressed in *Drosophila* [97].

Two important proteolytic pathways are the ubiquitin-proteasome pathway (UPP) and autophagy [98]. The UPP is a proteolytic system responsible for the majority of intracellular protein degradation. A key aspect of UPP-mediated proteolysis is the selective targeting of proteins for degradation via posttranslational modifications, particularly ubiquitination and sumoylation [77, 78]. Aging is associated with increased levels of ubiquitinated and sumoylated protein in various tissues [99–103], potentially as a result of age-dependent UPP malfunctioning [104, 105].

Ubiquitination pathways have been shown to play a significant role in regulating lifespan [106, 107]. In *Drosophila*, a loss-of-function mutation in the ubiquitin-activating enzyme Uba1 significantly reduced lifespan and weakened motor function [108]. In *C. elegans*, overexpression of the E3 ubiquitin ligase, WWP-1, increased lifespan via signaling mediated by the forkhead box A (FoxA) transcription factor [109].

Enhanced expression of the proteasome assembly protein Ump1 has also been associated with enhanced viability following exposure to various oxidative stress factors (e.g., menadione, hydrogen peroxide, and 4-hydroxynonenal) in *S. cerevisiae* [89]. This increased viability was associated with an enhanced preservation of proteasome-mediated protein degradation. Interestingly, cells expressing elevated levels of Ump1 also exhibited an enhanced preservation of proteasome-mediated protein degradation and enhanced viability during stationary-phase aging. Taken together, these data strongly support a key role of the proteasome during oxidative stress and aging [89].

Autophagy is also essential for maintaining protein homeostasis, as both cellular autophagy and mitophagy (autophagy of an entire mitochondrion) impact lifespan [90, 110]. Three autophagy proteins (LC3B, ATG5, and ATG12) play an important role in preserving mitochondrial integrity and lifespan [111]. In human umbilical vein endothelial cells, targeted mitochondrial damage was found to initiate a cascade of events involving a short-term increase in ROS production, followed by mitochondrial fragmentation and upregulation of LC3B, ATG5, and ATG12. This cascade significantly enhanced the replicative lifespan up to 150% and the number of population doublings up to 200% [111]. Additionally, in normal aging and during the progression of age-related pathologies, autophagy is responsible for the removal of proteins damaged by oxidation, for instance, from the brain to restore its proper function [112, 113].

During aging, mitochondria—the primary source of ROS—are often subjected to oxidative damage at a level that supersedes the protective capacity of the antioxidant response. In such cases, removal of damaged mitochondria through mitophagy is crucial to mitigate the detrimental effects on the organism [114]. Furthermore, in *C. elegans*, tight coupling between mitophagy and mitochondrial biogenesis is important for promoting longevity under stress conditions [115]. Also, in flies, overexpression of the mitophagy protein PARKIN has been shown to extend lifespan by enhancing mitochondrial turnover [116]. Therefore, mitophagy acts as a major marker of ROS-induced damage and plays a significant role in aging and various age-related disorders [117].

## 5. The Nucleus-Mitochondria Connection and the Importance of Mitochondrial Proteostasis

Nuclear DNA damage induces nuclear-to-mitochondrial signaling (NM signaling). This process plays a vital role in mitochondrial homeostasis and aging. Nuclear proteins (e.g., HIF-1*α*, proliferator-activated receptor gamma coactivator-1*α* (PGC-1*α*), forkhead box protein O (FOXO), and the sirtuin family) together with nuclear DNA damage repair proteins can affect mitochondrial integrity and contribute to age-related pathologies [118]. Recent studies have established an important connection between nicotinamide adenine dinucleotide (NAD^+^) and DNA repair proteins in maintaining mitochondrial metabolism and increasing lifespan [119].

Sirtuins, NAD^+^-dependent deacetylases, act as metabolic sensors that perceive imbalances in the NAD^+^/NADH ratio. The inhibition of DNA repair proteins, specifically NAD^+^-consuming poly (ADP-ribose) polymerase proteins (PARP-1 and PARP-2), increases cellular NAD^+^ levels [119]. High NAD^+^ levels subsequently activate sirtuins, which in turn promote higher mitochondrial content, increased energy expenditure, and protection against metabolic disease [119], ultimately extending longevity [120]. Furthermore, sirtuin activators, such as resveratrol, have been shown to promote longevity [121, 122] by inducing calorie restriction- (CR-) like effects in *C. elegans* [123].

However, both PARP and sirtuins must consume NAD^+^ to be functional. Large amounts of PARP and sirtuins can deplete cellular NAD^+^ levels. Depleted NAD^+^ levels lead to sirtuin inactivation and excessive ROS production, which alters mitochondrial integrity [124]. Moreover, perturbations in the activity of sirtuins deactivate several enzymes including PGC-1*α* (peroxisome proliferator-activated receptor gamma coactivator 1*α*), forkhead box O (FOXO) transcription factors, hypoxia-inducible factor-1*α* (HIF-1*α*), and AMP-activated protein kinase (AMPK), which modulates the production of various antioxidative enzymes, affecting oxidative defense mechanisms [125].

DNA-damage-induced NM signaling through the PARP-NAD^+^-sirtuin axis can accelerate the onset of aging by disrupting mitochondrial integrity. Thus, genetic or pharmacological interventions targeting proteins or metabolites involved in NM signaling can potentially promote longevity. For instance, in aging rats, treatment with the PARP inhibitor INO-1001 reduces cardiovascular disorders [126], and treatment with the PARP inhibitor PJ34 improves myocardial contractile function and restores endothelial function [127]. Furthermore, PARP-1 inhibition may protect against age-dependent endothelial dysfunction, potentially by regulating NO bioavailability via iNOS [128].

However, the beneficial role of PARP-1 inhibition in aging has been questioned [129]. For instance, PARP-1-null mice have a reduced lifespan, an earlier onset of aging, and an increased rate of spontaneous carcinogenesis compared with WT mice [130]. One explanation for discrepancies among studies is the dual role of PARP: while PARP contributes to maintain genomic stability and promote longevity, excessive PARP activity depletes cellular NAD^+^ and triggers nuclear factor-*κ*B- (NF-*κ*B-) induced inflammation, leading to the rapid onset of aging and age-related disorders [131].

**Figure 3:**
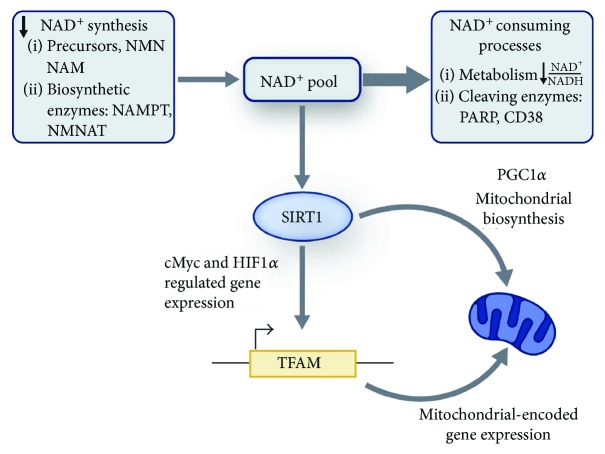
Age-dependent decline in NAD^+^. Decreased NAD^+^ synthesis and increased NAD^+^ consumption with age may both contribute to a decrease in the NAD^+^ pool. A reduction in NAD^+^ levels leads to an age-related reduction of SIRT1 activity. Reduced SIRT1 activity impacts mitochondrial function through at least two mechanisms: (1) a reduction in biogenesis secondary due to a reduction in PGC1-*α* activity and (2) an impairment of mitochondrial function due to a reduction in mtDNA replication and transcription. Reproduced with permission from Prolla, T.A. and Denu, J.M., 2014. NAD+ deficiency in age-related mitochondrial dysfunction. Cell Metabolism, 19(2), pp.178-180.

Aging is accompanied by decreased NAD^+^ synthesis and increased NAD^+^ consumption, resulting in a net decrease in the pool of available NAD^+^ (Figure 3). Reduced NAD^+^ levels lead to an age-related reduction of sirtuin 1 (SIRT1) activity. Reduced SIRT1 activity impacts mitochondrial function through at least two mechanisms: (1) reduced biogenesis secondary to a reduction in PGC1-*α* activity and (2) impaired mitochondrial function due to a reduction in mitochondrial DNA replication and transcription [132, 133]. Therefore, supplementation with NAD^+^ or its precursors is hypothesized to promote healthy aging and longevity [134–136].

Experimental models have shown that NAD^+^ supplementation is beneficial for maintaining carbohydrate metabolism, cardiovascular function, stem cell function, and longevity [137]. Moreover, nicotinamide prevents cellular senescence by reducing excessive ROS production [138, 139]. Several human clinical studies testing the efficacy of this compound are ongoing [140].

The NAD^+^-mediated improvement in *C. elegans* lifespan was shown to involve a series of interconnected mechanisms that include (1) activation of the worm sirtuin homolog Sir-2.1, (2) nuclear translocation and activation of the FOXO transcription factor *daf-16*, and (3) increased expression of antioxidative enzymes [141].

In a mouse model, treatment with the NAD^+^ precursor nicotinamide riboside (NR) delayed muscle and neural stem cell senescence and increased longevity. This effect seemed to be mediated by the induction of the mitochondrial unfolded protein response (UPRmt) [142]. Involvement of the UPRmt in the lifespan-extending effect of NAD^+^ has also been proposed in *C. elegans* [143].

The UPRmt is a form of retrograde signaling that contributes to ensuring the maintenance and functional integrity of the mitochondrial proteome [144]. Accumulation of misfolded proteins or unassembled complexes in the mitochondria beyond a certain threshold leads to altered proteostasis that can result in organelle/cell dysfunction [145]. Mitochondria relay this distress message to the cytosol and nucleus through various types of signals, and in response, the cell elicits a set of responses, including the production of mitochondrial localized molecular chaperones and proteases to promote the recovery of organellar protein homeostasis [91, 92, 146, 147].

An adaptive pathway triggered by a sirtuin-dependent UPRmt, which results in increased mitochondrial complex content and activity [143, 148], has been shown to lead to increased lifespan, at least in mice and flies [143, 142, 146]. Mitochondrial retrograde signaling to the nucleus via the mTOR pathway has also been found to extend normal human fibroblast lifespan, increase the mitochondrial membrane potential, reduce ROS level, and enhance autophagic flux [149]. ROS can exert an additional burden on the protein quality control system since protein chaperones themselves are susceptible to oxidative damage resulting in further damage accumulation and accelerated aging [4, 65].

Collectively, these studies establish a ROS-mediated connection between the mitochondria, the nucleus, and proteostasis.

## 6. Role of ROS in Nonpharmacological Strategies to Extend Lifespan

### 6.1. Calorie Restriction (CR)

The term “caloric restriction” designates reduced energy intake without malnutrition, and it represents the most effective and reproducible dietary intervention known to promote healthy aging and slow down the manifestation of age-related disorders in various model organisms including yeast [150–153], nematodes [154, 155], fruit flies [156], mice [157–159], and primates [160]. CR regulates numerous physiological processes associated with aging, including metabolism [161–165], oxidative stress [166, 167], genomic stability [168], and growth signals [169–171].

Four major theories have been proposed to account for the beneficial effects of caloric restriction. According to the “oxidative damage attenuation” hypothesis, oxidative damage is decreased during caloric restriction (CR), through the decreased production of reactive oxygen species and the upregulation of protective enzymes, resulting in a decrease in DNA damage and increase in genomic stability [168, 172, 173]. The “glucose-insulin” hypothesis suggests that the decreased levels of circulating insulin and glucose that accompany CR lead to decreased cell growth and division, shifting the resources of the cell towards maintenance and repair [172, 173]. The related “insulin-like growth factor (IGF) 1” hypothesis suggests that decreased levels of growth hormone and IGF-1 in response to CR promote maintenance and repair activities [172, 173]. Finally, the “stress-adaptation” (or hormesis) hypothesis suggests that CR promotes a low level of stress which induces cross-adaptation to other stress factors by increasing the levels of antioxidant and DNA repair proteins [174].

**Figure 4:**
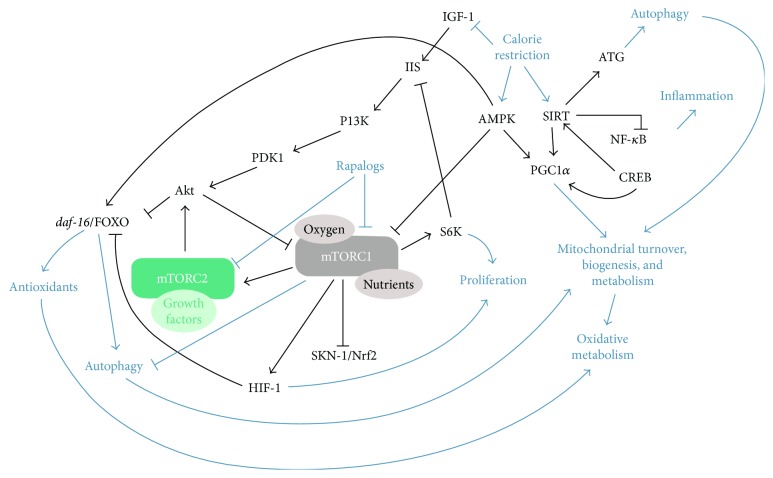
Crosstalk between mTOR and other longevity pathways. mTORC1 responds to a variety of environmental cues, including oxygen and nutrients, and communicates with several other known longevity factors in a complex network of interactions. Rapalogs inhibit mTORC and decrease its activity. Sensing of low oxygen levels stimulates mTORC1 to activate the hypoxic response by enhancing translation of HIF-1, which inhibits FOXO family members and increases longevity. mTORC1 inhibits the stress response transcription factor SKN-1/Nfr2, resulting in extended lifespan. Inhibition of the mTOR downstream effector ribosomal protein S6 kinase (S6K), involved in the regulation of protein translation, also results in extended lifespan. Caloric restriction can lower mTORC1 signaling partly through activation of AMPK, resulting in enhanced longevity, potentially via PGC1*α*-mediated increase in mitochondrial metabolism. Calorie restriction also inhibits IGF1-dependent signaling via PI3K/PDK1/Akt which inhibits FOXO, blocking the expression of antioxidants and autophagy. Calorie restriction leads to increased NAD^+^/NADH ratio, which activates sirtuins, that in turn induce mechanisms to enhance cell protection, including enhanced antioxidant production and autophagy. Calorie restriction can also block inflammation via the effects of sirtuins on NF-*κ*B. cAMP response element binding proteins (CREB) can also upregulate the transcription of sirtuins, slowing aging.

Several molecular explanations for the lifespan-extending effects of CR have been proposed. However, much is still unknown about the precise contribution of each pathway to the lifespan-extension effect of CR. This understanding is further complicated by the extensive crosstalk between the different pathways and by the fact that some pathways are present in some model organisms but not in others. The complex network of pathways that are involved in the lifespan-extending effects of caloric restriction is depicted in Figure 4.

Two of the most studied pathways purportedly involved in the lifespan-mediated extension conferred by CR are those mediated by inhibition of insulin/IGF-1 signaling and inactivation of mTOR (mechanistic target of rapamycin). Both are considered nutrient-sensing pathways (insulin for glucose and mTOR for amino acids). Decreases in circulating levels of nutrients (amino acids, glucose, and even cholesterol)—all of which are also sensed by mTOR—contribute to decreased mTOR activity during CR [175]. mTOR inhibition leads to SKN-1-/Nrf- and *daf-16*-/FOXO-mediated activation of protective genes, resulting in an increase in stress resistance and longevity [176]. Additionally, inhibition of mTOR is known to induce autophagy, which has an important role in proteostasis during aging [177, 178]. The lifespan-extending effect of mTOR inhibition, either genetically or chemically, seems to be very conserved across different model organisms [159, 179–181]. The insulin pathway is mediated via several additional enzymes including PI3K/Akt/Ras and the forkhead O (FOXO) transcriptional factor [182–184].

The pathway mediated by adenosine monophosphate-activated protein kinase (AMPK) is a third possible CR-relevant pathway that can, in some organisms, crosstalk with the mTOR pathway. AMPK is a highly conserved sensor of increased levels of AMP and ADP originating from ATP depletion [185–187]. In general, activation of AMPK acts to maintain cellular energy stores, switching on catabolic pathways that produce ATP, mostly by enhancing oxidative metabolism and mitochondrial biogenesis, while switching off anabolic pathways that consume ATP. The importance of AMPK in determining lifespan is demonstrated by the fact that treatment with metformin, an AMPK activator, extends the lifespan of *C. elegans* and short-lived, cancer-prone mice strains [188–190].

One additional important pathway is that directed by sirtuins, the activity of which increases with CR. Association of sirtuins with decreased oxidative stress levels and increased antioxidative defense has been proposed for several model organisms [191, 192], as well as humans, but the exact molecular mechanisms behind this association remain unclear. SIRT3 has been suggested as an essential player in enhancing the mitochondrial glutathione antioxidant defense system during caloric restriction [193]. SIRT3-dependent mitochondrial adaptation may also contribute to delaying aging in mammals [193].

Their role as mediators in the beneficial effects exerted by caloric restriction have made sirtuins promising pharmacological targets to delay aging and age-related diseases [194]. Resveratrol is a polyphenol antioxidant found in red wine and shown to activate sirtuins in several organisms, including humans [195, 196]. Resveratrol is also an AMPK activator, and this activity can also contribute to the beneficial effects of this polyphenol [197]. Purportedly, resveratrol upregulates antioxidant defense mechanisms and attenuates mitochondrial ROS production via sirtuin activation. Significant reduction of cellular hydrogen peroxide [198–200], upregulated MnSOD expression [195, 196], and increased cellular glutathione content [201] have been observed after resveratrol administration. The therapeutic potential of resveratrol has been the subject of intense research over the last decade (e.g., [195–198]).

CR has also been shown to reduce age-related accumulation of oxidative damage by decreasing mitochondrial respiration, membrane potential, and the rate of ROS production [166, 167], although CR seems to have only a minor effect on age-related changes in the mitochondrial proteome [202]. CR also increases mitochondrial biogenesis through the PGC-1*α* signaling pathway [203]. Moreover, other studies have also shown that CR protects from age-related vascular malfunctioning by increasing nitric oxide (NO) bioavailability, reducing ROS production, triggering anti-inflammatory responses, and preventing oxidative damage by activating the NRF-antioxidant response element (ARE) signaling pathway [204, 205].

Caloric restriction typically involves a 20–40% reduction of food consumption relative to normal intake. This is a rather severe intervention that can have detrimental effects [191]. Intermittent or periodic dietary restrictions without chronic caloric restriction have the potential to provide a significant health span increase while minimizing adverse effects. In fact, studies in rodents have shown that even a 10% decrease in food consumption can substantially affect lifespan [206]. *Sod*^−/−^ mice show increased levels of oxidative stress, which in turn results in reduced lifespan. Dietary restriction (60% of ad libitum fed diet) was shown to increase the lifespan of *Sod*^−/−^ mice by 30%, making it similar to that of wild-type, control mice fed ad libitum [207], by reducing lipid peroxidation in the liver and brain. The same dietary intervention was found to attenuate age-associated muscle atrophy by lowering oxidative stress in mice even in complete absence of the key antioxidant enzyme CuZnSOD [208].

### 6.2. Exercise

Exercise is another effective nonpharmacological means of delaying the negative effects of aging. Several studies reported elevated O_2_ load in skeletal muscle fibers [209, 210] and increased ROS levels [209, 211] during exercise as a result of increased mitochondrial respiration required to generate ATP for muscle contractions. While mitochondrial oxidative phosphorylation is the primary source of exercise-induced ROS, xanthine oxidase and endothelial nitric oxide synthase (eNOS) also contribute to ROS generation during endurance training [212] and stretching exercises [40, 41]. Regular exercise has been associated with lowered mortality and incidence of age-related diseases [213–215]. Therefore, exercise interventions potentially could have benefits for older individuals through modulation of inflammatory and redox status, which can influence proteostasis, insulin sensitivity, body composition (e.g., adipose tissue), and hormone levels [216].

An aging-associated increase in ROS production in skeletal and cardiac muscle cells during rest and in a postexercise state has been reported [211, 217]. At the muscular level, age-related increases in ROS levels have been associated with various mechanisms, such as ETC dysregulation due to decreased activity of cytochrome c oxidase and other enzymes [218] and mitochondrial membrane disruption due to lipid peroxidation and unsaturation [44, 45].

However, conflicting results also have been reported. A study of the skeletal and cardiac muscle tissues of aged rats showed a significant increase in antioxidant enzymes, such as SOD, catalase, GPX [47–50], and glutathione (GSH) [51, 132]. Additionally, muscles that undergo chronic exercise show lower oxidative stress in terms of lipid, protein, and DNA damage in both humans and model organisms [38, 49, 133]. Accordingly, mitochondria isolated from trained muscle cells showed higher oxidative resistance *in vitro* [55, 219]. Studies also show an increase in SOD, GPX, and GSH levels following endurance training in both young and old individuals [56, 57]. These results suggest that regular physical exercise is accompanied by an adaptation of the cells to deal with oxidative stress, which in turn elicits beneficial effects, for example, in the immune system [220]. This idea is summarized by the concept of hormesis. Hormesis can be defined as the adaptive response seen in organisms continuously exposed to low to moderate levels of stress. Under these conditions, cells develop an adaptive response, including increased expression of antioxidant genes, which in turn makes them resistant to multiple stressors [221, 222].

The induction of hormesis is controlled by redox sensor pathways which, upon activation by oxidants, upregulate the antioxidant enzymatic system [223]. For example, intense physical exercise activates the mitogen-activated protein kinase (MAPK) and the NF-*κ*B redox signaling pathways in both humans and rodents [60, 61]. The major targets of these pathways are antioxidant enzymes, including SOD, GPX, and GSH which contain NF-*κ*B and activator protein-1 (AP-1) binding sites in their promoters [62–65] as well as responsive elements to various stimuli like proinflammatory cytokines, oxygen tension, and ROS [66–68]. In skeletal muscles, another crucial hormetic adaptation to oxidative stress is the increase in mitochondrial mass and protein content [69, 70], particularly the level of cytochrome c oxidase. Cytochrome c oxidase controls electron flow and the superoxide formation in the ETC [224]. These changes upregulate the expression of PGC-1, which drives mitochondrial biogenesis in skeletal muscles during exercise [225]. PGC-1 is also linked with reduced oxidative stress [226].

It has been hypothesized that this hormetic response to oxidative stress becomes impaired as skeletal muscles age [218, 227]. This hypothesis is supported by several studies reporting significantly lower NF-*κ*B expression and activity in aged muscles [217, 228, 229]. By contrast, other studies have reported unchanged [230] or even higher [231] NF-*κ*B levels at a resting state and decreased MAPK pathway activation postexercise in aged muscles of rodents and humans.

While exercise interventions have been proposed as effective, nonpharmacological means of delaying the negative effects of aging on functional and metabolic parameters [216], it is also well known that regular vigorous exercise can have detrimental effects, as evidenced by the enhanced susceptibility of elite athletes to infections [232]. This effect seems to be at least partly due to the detrimental effects of long-term exposure to the enhanced ROS production associated with intense exercise practice. For instance, chronic muscle injury, a common affliction of not only athletes but also older individuals, increases the production of proinflammatory cytokines, such as tumor necrosis factor-*α* (TNF-*α*) and interleukin-6 (IL-6), which further contribute to oxidative stress that, in turn, exacerbates muscle inflammation, creating a vicious cycle of inflammation and oxidative damage [233].

Additional studies are needed to resolve the conflicting results regarding the effects of exercise and exercise-induced hormesis on the oxidative stress status skeletal muscles and its progression throughout the lifespan.

## 7. ROS versus Aging: May Bacteria Take the Stand

Until recently, dogma held that bacteria do not undergo any events that are equivalent to the aging process [234]. However, this viewpoint has changed over the last decade. Bacterial aging was first reported in the asymmetrically dividing *Caulobacter crescentus* [235, 236]. In this *α*-Proteobacteria, cell division is both morphologically and functionally asymmetric. This asymmetry produces a clear distinction between mother and daughter cells. Ackermann et al. [162] reported that, over multiple divisions, the time required for a mother cell to yield a new daughter cell doubled from 2.6 h to 5 h per division cycle, a process similar to replicative aging in eukaryotes [235, 236]. Later, Stewart et al. [237] demonstrated that *Escherichia coli* also displayed features of replicative aging despite dividing by symmetrical, binary fission. Using automated time-lapse microscopy to image 8–10 reproduction cycles of individual cells, the authors observed that the old-pole (mother) cells showed a decreased growth fitness (e.g., growth rate) over successive generations, compared with their new-pole (sister) cells. The old-pole cells had reduced offspring formation and increased incidence of cell death. After approximately 100 divisions, the old-pole cells ceased to grow [237].

**Figure 5:**
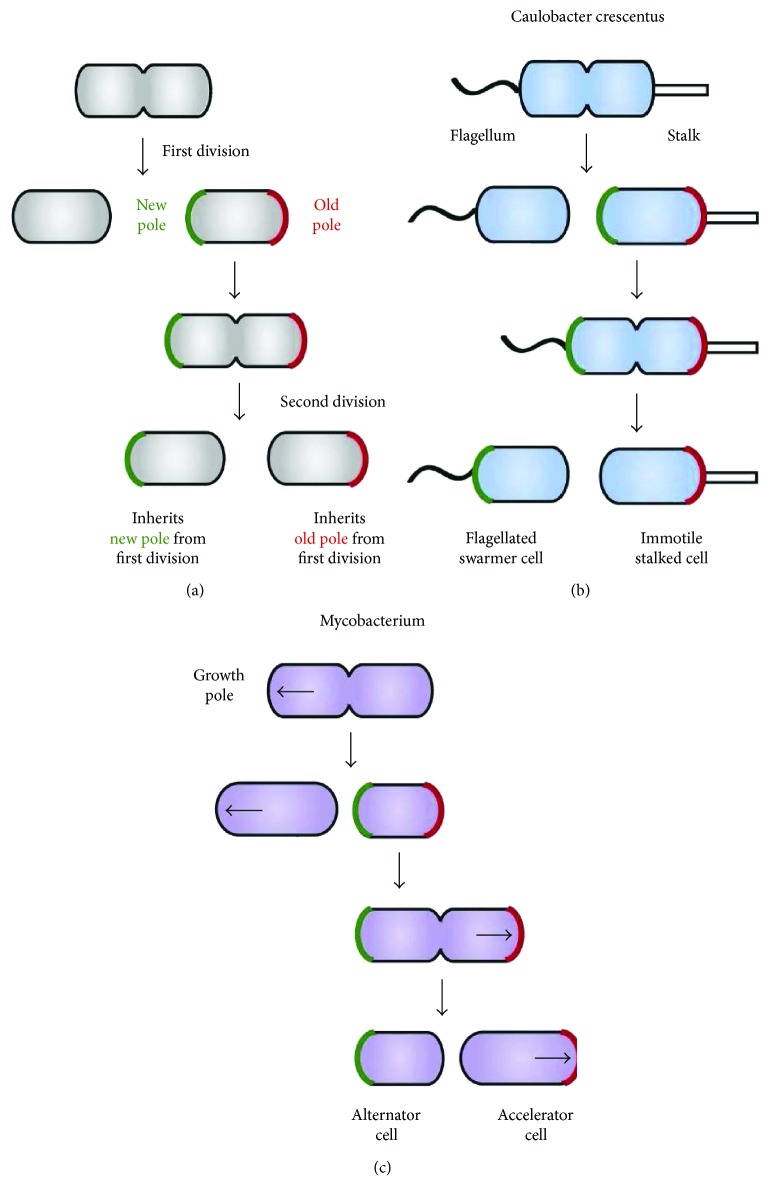
(a) All cell divisions in rod-shaped bacteria are asymmetric in that one daughter cell inherits the “new” pole (green) from a previous division and the other inherits the “old” pole (red). In some bacteria, this asymmetry is used to create functional specialization of daughter cells. (b) In *C. crescentus*, different polar appendages form at the new and old poles, leading to dimorphic daughter cells. (c) In *Mycobacterium*, cells preferentially grow at the old pole (marked with an arrow). Daughter cells that inherit the old pole, called accelerators, continue growing whereas those inheriting the new pole, called alternators, must form a new growth pole before elongating. Reproduced with permission from Aakre CD, Laub MT. Asymmetric cell division: a persistent issue? Developmental cell. 2012; 22 (2):235-236. doi:10.1016/j.devcel.2012.01.016.

Subsequent research demonstrated that similar processes occur in *Bacillus subtilis* [238] and *Mycobacterium* spp. [239] (Figure 5). These observations confirm that aging in bacteria is a more general phenomenon than once thought, which affects not only microbes with distinct morphologies within the mother-to-daughter lineage but also those in which growth asymmetry is seen in the progeny at the functional/molecular level.

### 7.1. Aging and Conditional Senescence

During the stationary phase, as a result of nutrient limitation, *E. coli* cells enter a unique state known as conditional senescence [240]. Once rendered senescent, bacteria continuously lose their culturability and are unable to resume growth even when nutrients become available again. This feature makes conditional senescence very similar to human somatic cell senescence [100–103], and the replicative lifespan of yeast (*S. cerevisiae*), which is commonly used to model the aging process of mitotic tissues in higher organisms [241].

The observed functional asymmetry in bacterial division, initially reported by Stewart et al. [237], has been associated with asymmetric segregation of damaged cell components (e.g., protein aggregates) [242, 243], a process also present in eukaryotes [244–246]. Asymmetric protein damage aggregation seems to be an active process in yeast [87]. In bacteria, this process seems to be mainly passive and driven by molecular crowding [247].

**Figure 6:**
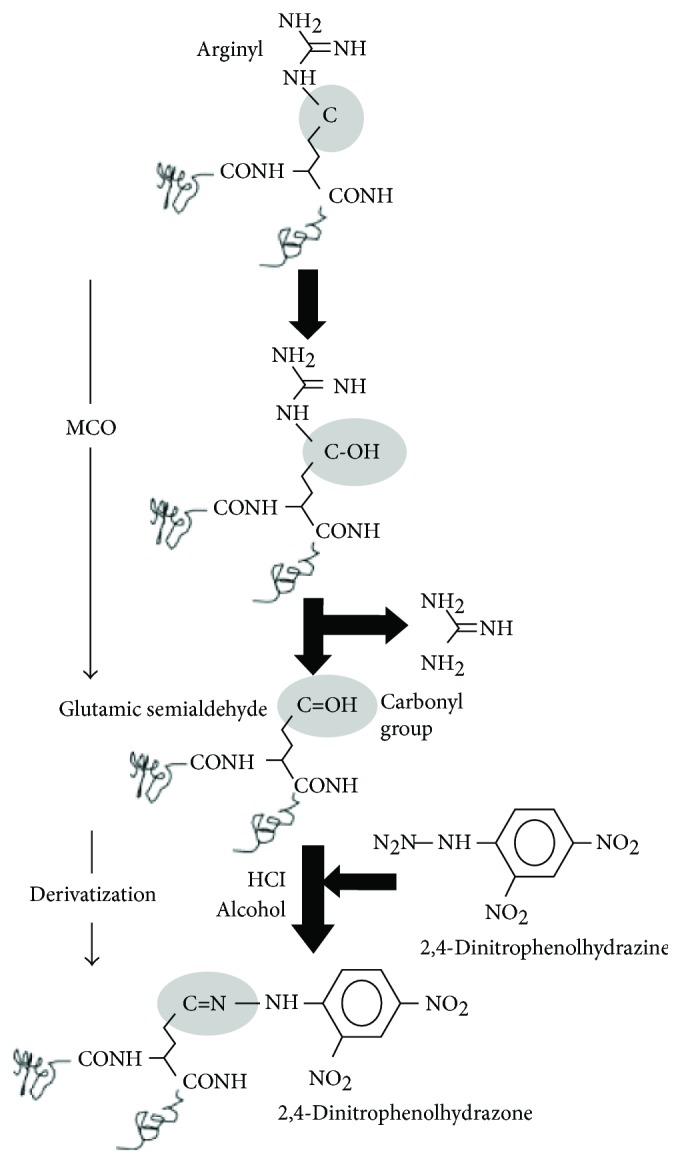
Carbonylation and derivatization of a protein amino acid side chain. A scheme for the formation of glutamic semialdehyde from an arginyl residue is depicted as a consequence of an MCO. For detection, the carbonyl group, in this case, glutamic semialdehyde, is subsequently derivatized by 2,4-dinitrophenolhydrazine. The resulting protein 2,4-dinitrophenolhydrazone can be detected by specific monoclonal or polyclonal antibodies [210]. Reproduced with permission from Nyström T. Role of oxidative carbonylation in protein quality control and senescence. The EMBO Journal. 2005; 24 (7):1311-1317. doi:10.1038/sj.emboj.7600599.

Batch cultures of *E. coli* subjected to starvation-induced growth arrest exhibit markedly higher loads of damaged (carbonylated) proteins [248], a feature also present in aging eukaryotes [249, 250]. However, this load does not seem to be uniformly distributed in the population. Interestingly, low-carbonyl-load cells remained reproductively competent, whereas high-carbonyl-load cells were genetically dead (i.e., unable to be cultured). Whether this starvation-induced heterogeneity in carbonylation and fitness is programmed and whether it is the result of damage segregation during cytokinesis has not been elucidated. Bacterial cell senescence induced by other external stimuli, including UVA radiation, is also associated to the accumulation of protein carbonyls as a result of oxidative damage [251–253] (Figure 6).

**Figure 7:**
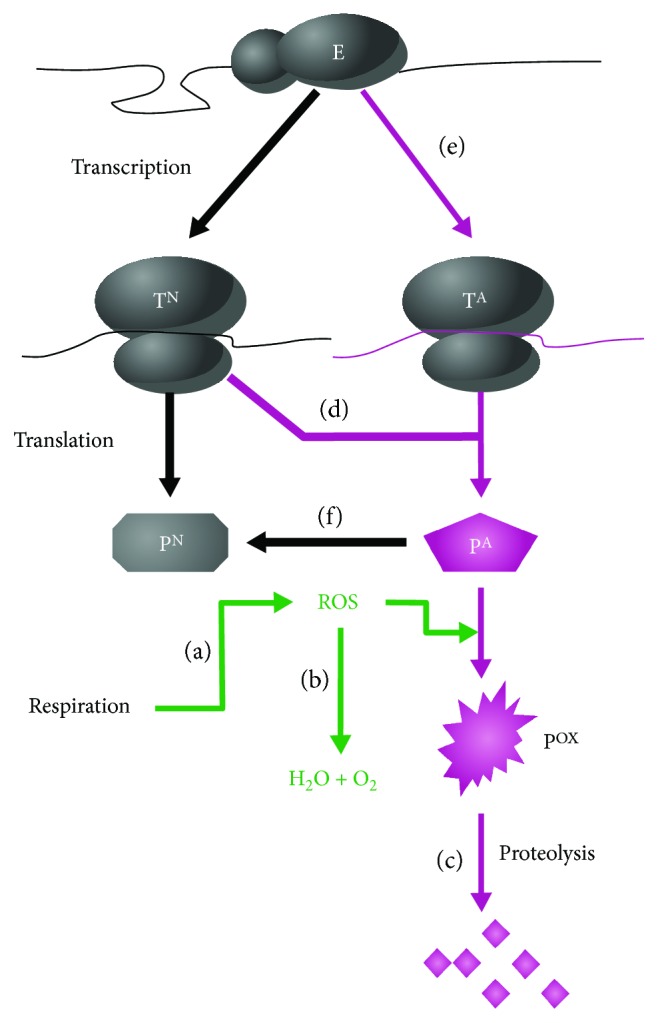
Activities of potential importance for stasis-induced oxidation of proteins. Traditionally, increased protein oxidation has been argued to be an effect of (a) increased production of reactive oxygen species (ROS), presumably derived from respiratory activity, (b) diminished activity or abundance of the antioxidant systems, or (c) reduced activity of the proteolysis or damage repair systems. Work on *E. coli* has highlighted the role of some alternative pathways in protein oxidation. These pathways relate to the production of aberrant proteins, which are highly susceptible to oxidative modification (carbonylation). Increased levels of such aberrant, malformed polypeptides can be the result of (d) reduced translational fidelity, (e) reduced transcriptional fidelity, or (f) diminished activity of the repair refolding apparatus. In the early stages of *E. coli* growth arrest, reduced translational fidelity appears to be the most important contributing factor to the elevated levels of oxidatively modified aberrant proteins. E, core RNA polymerase; P^A^, aberrant protein; P^N^, native protein; P^ox^, oxidized protein; T^A^, aberrant transcript; T^N^, native transcript. Reproduced with permission from Nyström, Thomas. “Aging in bacteria.” Current Opinion in Microbiology 5, no. 6 (2002): 596-601.

Time-dependent accumulation of protein carbonyls has been observed during the stationary phase in *E. coli* [254]. The activities that contribute to protein oxidation during the stationary phase are shown in Figure 7. Given that one of the criteria for aging is an increase in mortality rate over time [255], this time-dependent accumulation of protein carbonyls provides a compelling argument that prokaryotes, such as *E. coli*, age. Some proteins, such as tricarboxylic acid (TCA) cycle enzymes, seem to be particularly susceptible to carbonylation [256]. Interestingly, cells lacking SOD-1 activity display higher amounts of protein carbonylation and lose viability more rapidly in the stationary phase [248]. Furthermore, stationary-phase populations incubated in the absence of oxygen have significantly extended lifespans compared to counterparts grown in the presence of oxygen [254]. These observations highlight the involvement of ROS and oxidative stress in stationary phase-associated senescence.

During the stationary phase and under stressful conditions, the oxidation of specific proteins in *E. coli* takes place. These proteins include DnaK (an Hsp70 chaperone), DNA-binding protein H-NS, universal stress protein A (UspA), the elongation factors EF-Tu and EF-G, glutamine synthetase, glutamate synthase, and aconitase [254, 256, 257]. Interestingly, some of these proteins are also carbonylated in yeast cells under oxidative stress [258], in aging flies [259, 260], and in the human brain of individuals with Alzheimer's disease [261]. These observations suggest that unchecked oxidative damage in the form of protein carbonylation could be the proximal cause of aging among stationary-phase *E. coli* populations [248]. However, there is no direct proof of this hypothesis.

Growth-arrested, stationary-phase *E. coli* develop resistance to heat and oxidative stress, a phenomenon known as stasis-induced cross-protection [262]. Cells starved of carbon or nitrogen are markedly more resistant to heat shock and oxidative stress than proliferating cells [262, 263]. An association between stress resistance and lifespan has been described in several eukaryotic model organisms [264–267]. These observations indicate that there might be an evolutionarily conserved mechanism channeling resources away from reproduction and toward maintenance and protective functions [268]. Similar to eukaryotes, the ability of cells to quench ROS may play a role in determining the bacterial lifespan [248].

However, as with eukaryotes, there are conflicting results regarding the contribution of ROS to bacterial senescence. For instance, reproductively arrested populations of *E. coli* have increased levels of oxidative defense proteins and increased population resistance to external oxidative stresses [138, 139]. However, these populations also display higher levels of damaged proteins [254, 256]. Additionally, no strict correlation has been observed between respiratory activity, protein oxidation, and the lifespan of growth-arrested *E. coli* [269]. Similar results have been observed in G0-growth-arrested yeast cells [270].

The first genes induced following growth arrest in bacteria play roles in countering stasis-induced senescence and death [262, 271]. Many of these genes encode proteins that protect the cell from external stresses, such as heat, oxidants, and osmotic challenge, which could account for stasis-induced cross-protection [262]. Cross-protection relies on the sigma factor Sigma-S [272]. Under not only starvation but also general stress conditions, the Sigma-S transcription factor accumulates, binds, and directs RNA polymerases toward more than 50 specific genes [272]. *E. coli* mutants lacking Sigma-S have elevated levels of proteins with oxidative damage [254, 256] and accelerated senescence during growth arrest [272]. In *Salmonella* sp., both Sigma-S and Sigma-E are required for protection against oxidative damage in the stationary phase and mutants lacking Sigma-E have reduced survival and increased susceptibility to oxidative stress [273]. However, under anaerobic stationary-phase conditions, survival is completely preserved [273], indicating that oxidative injury is a major mechanism by which microbial viability is reduced during nutrient deprivation. Interestingly, members of the Sigma-S regulon include a diverse set of proteins with functions that overlap those of FOXO-/*daf-16*-regulated longevity genes in *C. elegans* [142, 147, 148]. Thus, functionally similar signaling pathways seem to regulate stress resistance, protein damage protection, and longevity in eukaryotes and prokaryotes. These pathways are pivotal for survival during periods of starvation. They may have been evolutionarily conserved across different branches of the tree of life because they enhanced the maintenance capacity of the cell. Over time, they also may have become crucial for retarding aging in multicellular organisms [143, 149].

### 7.2. Genetic Determinants of Senescence and Aging in Bacteria

Literature investigating the genes that extend stationary-phase survival in bacteria is scarce. However, a few mutant strains that survive longer than WT have been reported. RssB, which regulates the stability of the sigma factor Sigma-S, has been found to play a key role in the survival of *E. coli*, potentially by increasing the cell's resistance to spontaneous, endogenous stresses [274].

More recently, a genome-wide screen for *E. coli* mutants with a prolonged stationary-phase survival phenotype identified three strains that lived longer than WT [275]. One of the strains, Δ*sdhA* (succinate dehydrogenase subunit A), displayed increased stress resistance and extended lifespan. Succinate dehydrogenase is a tetrameric protein complex that catalyzes the conversion of succinate to fumarate in the TCA cycle [276]. Subunit A, the enzymatically active part of the complex, is a well-established source of superoxide in the ETC of *E. coli* [277]. Purportedly, when this enzyme is absent, the rate of superoxide production is reduced, extending stationary-phase survival [275].

The two other mutants displaying extended stationary-phase survival were Δ*lipA* (lipoyl synthase) and Δ*lpdA* (dihydrolipoyl dehydrogenase) [275]. The authors attributed the enhanced lifespan of these two mutants to their reduced consumption of oxygen, compared to WT, which in turn increased the expression of the hypoxia transcription factor ArcA [278]. ArcA suppresses the expression of TCA cycle genes, such as citrate synthase (*gltA*), and activates the expression of genes required to generate energy under oxygen-limited conditions, extending stationary-phase survival [279]. These observations suggest that the extended lifespan observed in these mutants is associated with the induction of a physiological state typically associated with hypoxic conditions. These results are consistent with the lifespan-modulating role of HIF-1*α* in higher organisms [37, 162, 163]. In fact, ArcA could be considered a functional homolog of HIF-1*α*, although the two proteins do not share significant sequence similarity. This functional similarity points toward the adaptive response to oxygen-limited conditions as an evolutionarily conserved mechanism that can extend lifespan.

Given the conservation of key phenotypes associated with age-dependent macromolecular damage and the lifespan-extending role of genes that control the hypoxic response in both bacteria and higher eukaryotes, it is reasonable to hypothesize that the most fundamental mechanisms of aging might be conserved at all levels of life. Future studies will help to clarify what molecular processes underlying aging are similar between bacteria and eukaryotes. The results of these studies could open the possibility of using *E. coli* as a model organism of aging on which specific molecular mechanisms and evolutionary theories can be easily tested.

## 8. Conclusions and Future Perspectives

The progressive loss of mitochondrial function is a consistent and conserved hallmark of aging that impacts both cellular homeostasis and organismal health [134, 135]. While ROS contribute to aging, they also play a crucial role in cell signaling and development, thus serving a beneficial role. The mitochondrial theory of aging offers a conciliatory perspective of the dual role of ROS in the aging process by incorporating two important adaptive responses: (1) UPRmt-mediated retrograde signaling from the mitochondria to the nucleus to regulate aging and (2) ROS-mediated adaptive response to activate the antioxidant defense system of the cell. Interventions targeting either of these two adaptive pathways could be considered potential targets for antiaging and lifespan-promoting therapies.

Because the accumulation of oxidative damage throughout life is a major cause of aging, genetic or pharmacological interventions targeting oxidative damage repair or damage removal pathways themselves also have significant therapeutic potential. However, further research in humans and nonhuman primates is needed to gain insights into the clinical significance of potential genetic, pharmacological, and nonpharmacological interventions.

The observation that several of the processes that characterize eukaryotic aging can also be seen in bacteria highlights the potential of bacteria to serve as a simple model organism to study aging and age-related mechanisms. These tractable models might provide crucial assistance in the quest to uncover the genetic, molecular, and biochemical processes underlying aging and age-related diseases.
